# PM2.5 IS ASSOCIATED WITH DAILY OUTPATIENT VISITS FOR ANKYLOSING SPONDYLITIS: A TIME-SERIES ANALYSIS IN CHINA

**DOI:** 10.1093/geroni/igae098.2823

**Published:** 2024-12-31

**Authors:** Hongbo Chen

**Affiliations:** Peking University Third Hospital, Beijing, Beijing, China (People’s Republic)

## Abstract

Exposure to ambient fine particulate matter (PM2.5) has a great impact on human body’s immune system, but the correlation between PM2.5 and ankylosing spondylitis has not yet been clarified. We extracted 58,600 outpatient visits for ankylosing spondylitis from the Beijing Medical Claim Data for Employees database from 2010 to 2017. The percentage of outpatient visits following PM2.5 concentrations was estimated using generalized additive models with Poisson connections. Increase by 10 μ g/m3, PM2.5 is associated with daily outpatient visits for ankylosing spondylitis. In this test, the average concentration of PM2.5 was 86.8 (74.3) μ g/m3. For every 10 μg/m3 increase in PM2.5 concentration, there was a 0.34% (95% CI, 0.26-0.42%) increase in the risk of patients who visited the doctor on the same day. Females and younger patients were most susceptible to the impact of PM2.5 exposure (P<0.05). This study revealed the relationship between exposure to PM2.5 and ankylosing spondylitis, and future research can further confirm this finding and explore the potential mechanisms.

